# Tripartite motif-containing protein 21 is involved in IFN-γ-induced suppression of hepatitis B virus by regulating hepatocyte nuclear factors

**DOI:** 10.1128/jvi.00468-24

**Published:** 2024-05-23

**Authors:** Juhee Won, Hong Seok Kang, Na Yeon Kim, Mehrangiz Dezhbord, Kamindu Gayashan Marakkalage, Eun-Hwi Lee, Donghyo Lee, Soree Park, Dong-Sik Kim, Kyun-Hwan Kim

**Affiliations:** 1 Department of Precision Medicine, School of Medicine, Sungkyunkwan University, Seoul, Republic of Korea; 2 Department of Pharmacology, Center for Cancer Research and Diagnostic Medicine, IBST, School of Medicine, Konkuk University, Seoul, Republic of Korea; 3 Department of Surgery, Division of HBP Surgery and Liver Transplantation, Korea University College of Medicine, Seoul, Republic of Korea; University of Southern California, Los Angeles, California, USA

**Keywords:** hepatitis B virus, tripartite motif-containing protein 21, interferon-gamma, hepatocyte nuclear factor

## Abstract

The antiviral role of the tripartite motif-containing (TRIM) protein family , a member of the E3-ubiquitin ligase family, has recently been actively studied. Hepatitis B virus (HBV) infection is a major contributor to liver diseases; however, the host factors regulated by cytokine-inducible TRIM21 to suppress HBV remain unclear. In this study, we showed the antiviral efficacy of TRIM21 against HBV in hepatoma cell lines, primary human hepatocytes isolated from patient liver tissues, and mouse model. Using TRIM21 knock-out cells, we confirmed that the antiviral effects of interferon-gamma, which suppress HBV replication, are diminished when TRIM21 is deficient. Northern blot analysis confirmed a reduction of HBV RNA levels by TRIM21. Using Luciferase reporter assay, we also discovered that TRIM21 decreases the activity of HBV enhancers, which play a crucial role in covalently closed circular DNA transcription. The participation of the RING domain and PRY-SPRY domain in the anti-HBV effect of TRIM21 was demonstrated through experiments using deletion mutants. We identified a novel interaction between TRIM21 and hepatocyte nuclear factor 4α (HNF4α) through co-immunoprecipitation assay. More specifically, ubiquitination assay revealed that TRIM21 promotes ubiquitin-mediated proteasomal degradation of HNF4α. HNF1α transcription is down-regulated as a result of the degradation of HNF4α, an activator for the HNF1α promoter. Therefore, the reduction of key HBV enhancer activators, HNF4α and HNF1α, by TRIM21 resulted in a decline in HBV transcription, ultimately leading to the inhibition of HBV replication.

**IMPORTANCE:**

Despite extensive research efforts, a definitive cure for chronic hepatitis B remains elusive, emphasizing the persistent importance of this viral infection as a substantial public health concern. Although the risks associated with hepatitis B virus (HBV) infection are well known, host factors capable of suppressing HBV are largely uncharacterized. This study elucidates that tripartite motif-containing protein 21 (TRIM21) suppresses HBV transcription and consequently inhibits HBV replication by downregulating the hepatocyte nuclear factors, which are host factors associated with the HBV enhancers. Our findings demonstrate a novel anti-HBV mechanism of TRIM21 in interferon-gamma-induced anti-HBV activity. These findings may contribute to new strategies to block HBV.

## INTRODUCTION

Hepatitis B virus (HBV) infection leading to chronic hepatitis B (CHB), cirrhosis, and hepatocellular carcinoma (HCC) remains a major global public health concern, affecting 297 million people chronically (1). Although pegylated interferon-alpha (PEG-IFNα), which regulates antiviral immune responses and nucleos(t)ide analogs, which targets the reverse transcriptase (RT) domain of HBV polymerase to inhibit HBV DNA synthesis, has been approved and administered for standard of care medication for CHB, it cannot induce a functional cure for majority of CHB patients (2). To achieve a functional cure for CHB, understanding the relationship between HBV replication and host antiviral factors is essential.

Following HBV infection of hepatocytes via sodium-taurocholate co-transporting polypeptide (NTCP), relaxed circular DNA enters the nucleus and undergoes a multi-step process with host machineries to convert into covalently closed circular DNA (cccDNA) (3). Transcription from cccDNA is regulated by host factors, including liver-enriched transcription factors such as hepatocyte nuclear factors (HNFs) and CCAAT/enhancer binding protein (C/EBP), binding to HBV enhancer I (EnhI) and II (EnhII) regions (4). Notably, HNF1α and HNF4α play crucial roles in hepatocyte differentiation and maintenance (5) and are decisive for cccDNA transcription (6). The expression of HNFs is mainly regulated by mitogen-activated protein kinase (MAPK) signaling pathways (7, 8), and the host factors promoting their expression or enhancing stability are not well understood.

Tripartite motif proteins (TRIMs), with over 80 members in humans, serve various functions in differentiation, proliferation, gene expression, damage repair, apoptosis, inflammation, and immunity (9–15). The RING domain, present in almost all TRIM family proteins, confers the catalytic activity of E3 ligases, with ubiquitination associated with this domain being a significant role (16). For instance, TRIM52 restricts Japanese encephalitis virus replication through the ubiquitination of JEV nonstructural protein 2A (NS2A), leading to proteasomal degradation (17). TRIM31 catalyzes K63-linked poly-ubiquitination of lysines on mitochondrial antiviral-signaling protein (MAVS), promoting aggregation and activation of the signaling adaptor MAVS to elicit antiviral response (18). The PRY/SPRY domain, found at the C-terminus of most TRIMs, can interact with RNA or proteins, and some exhibit Fc receptor-like activity (10, 13). TRIM5α, binding to HIV capsid via the SPRY domain, restricts it through the innate immune system (19). TRIM25 interacts with retinoic acid-inducible gene I (RIG-I) and enhances the recognition of HBV pregenomic RNA (pgRNA), further promoting interferon production (20).

The E3-ubiquitin ligase tripartite motif-containing protein 21 (TRIM21), initially identified as autoantigen Ro52/SS-A1 in autoimmune diseases, was later recognized as an E3 ligase and Fc receptor and subsequently termed TRIM21 (21). TRIM21 acts as an antibody-binding protein in innate immune cells and functions as a cytosolic Fc receptor regulating viral infection (22). The previous study identified a cell-penetrating antibody targeting hepatitis B virus X protein (HBx), which induces TRIM21-dependent degradation (23). TRIM21 targets HBx for ubiquitin-dependent proteasomal degradation, thereby restricting HBV replication (24). Despite these studies, the host factors regulated by TRIM21 that participate in the antiviral activity against HBV are still largely unknown.

In this study, we revealed that TRIM21 restricts HBV replication by regulating HNFs through the ubiquitin-proteasome pathway.

## MATERIALS AND METHODS

### Cell culture and transfection

Human hepatoma cell lines Huh7 and HepG2 were purchased from the American Type Culture Collection and cultured in Dulbecco’s modified Eagle’s medium (DMEM; Welgene, Gyeongsan-si, Korea) supplemented with 10% fetal bovine serum (FBS; Capricorn, Ebsdorfergrund, Germany) and 1% penicillin/streptomycin (Gibco, Grand Island, NY, USA) at 37°C in a 5% CO_2_ incubator. The HepAD38 cell line, which stably produces HBV particles, was cultured in the presence or absence of tetracycline (0.3 µg/mL). TRIM21 knock-out cell line HepG2-TRIM21 KO was generated as described in this Materials and Methods section and maintained in DMEM containing 10% FBS, 1% penicillin/streptomycin, and puromycin (5 µg/mL; Sigma-Aldrich, Saint Louis, MO, USA). Primary human hepatocytes (PHHs) were cultured in cell maintenance supplements (CM4000; Gibco, Grand Island, NY, USA) and 1% penicillin-streptomycin was added to William’s medium E (Gibco, Grand Island, NY, USA). To transfect the cells, Lipofectamine 2000 (Invitrogen, Carlsbad, CA, USA) for Huh7, HepG2, and HepG2-NTCP cell lines and Lipofectamine 3000 (Invitrogen, Carlsbad, CA, USA) for PHHs were used according to the manufacturer’s instructions

### Plasmids and reagents

The plasmids for replication-competent HBV genotype D constructs, WT HBV 1.2mer [pHBV1.2(+)], X-null HBV 1.2mer [pHBV1.2(−)], pHBx-HA, pUb-HA, and HBV enhancer-luciferase reporters were used in our previous study (25). PCR was performed using the cDNA library of Huh7 as a template, and the forward primer ccggaattcgggcttcagcagcacgcttgac and reverse primer ccgctcgagtcaatagtcagtggatccttg were used for the TRIM21 gene. PCR products of the TRIM21 gene were obtained and inserted into the pCMV-Myc vector (Clontech, Mountain View, CA, USA) to subclone pMyc-TRIM21. Likewise, cloning the wild-type TRIM21 plasmid, the forward primer sequence cccgaattcgctttctgctcaagaatctcc and reverse primer sequence same as wild type were used for deletion mutation of TRIM21 in the RING domain deletion mutant. For the primer that made PRY-SPRY domain deletion mutants, the forward was the same as wild type, and the reverse sequence was ccgctcgagtcaacatgtcctcagcatctt. RING and PRY-SPRY, both domain deletion mutants, were created using the same forward primer for RING deletion and the same reverse primer for PRY-SPRY deletion. PCR was performed using the Huh7 cell cDNA to obtain the PCR product of each mutant, followed by pCMV-Myc vector subcloning. pLentiCRISPR V2-TRIM21 (GenScript, Piscataway, NJ, USA), pCMV delta R8.2 (#12263; Addgene, Watertown, MA, USA), and pCMV-VSV-G (#8454; Addgene, Watertown, MA, USA) were purchased. To generate the mutant HBV enhancer I luciferase reporter where HNF4α binding site was deleted (EnhI-ΔHNF4α), PCR was performed using pHBV1.2(+) as a template with the following primers: forward primer 1: tctatcgataggtacctcctattaacaggcctattgattggaaag, reverse primer 1: aggtattgtttacacagaaaggcc, forward primer 2: tgtgtaaacaatacctccccgttg cccggcaacggccaggtctgtgccaagtg, and reverse primer 2: cggaatgccaagcttacttagatcgca gatctggtccggcagatgagaag. Afterward, the created insert was cloned into the pGL3-basic vector. Primary antibodies against proteins and tags indicated in the figures were used: TRIM21 (sc-48430; Santa Cruz Biotechnology, Dallas, TX, USA), HNF4α (H-1; Santa Cruz Biotechnology, Dallas, TX, USA), HNF1α (F-7; Santa Cruz Biotechnology, Dallas, TX, USA), HNF3β (RY-7; Santa Cruz Biotechnology, Dallas, TX, USA), HBx (Biovendor, Heidelberg, Germany), Myc (ab39688; Abcam, Cambridge, UK), HA (H6908; Sigma-Aldrich, Saint Louis, MO, USA), interleukin 32 (IL-32) (PAB101, YbdY, Seoul, Korea), and β-actin (A5441; Sigma-Aldrich, Saint Louis, MO, USA). Reagents used in this study included human IFN-γ (LG, Jeonbuk, Korea), human tumor necrosis factor-alpha (TNF-α) (YbdY, Seoul, Korea), U0126 (Cell Signaling, Boston, MA, USA), and MG132 (Sigma-Aldrich, Saint Louis, MO, USA).

### Luciferase assay

HepG2 or Huh7 cells (approximately 2.5 × 10^5^ cells/well) were seeded on 12-well plates and co-transfected with EnhI, EnhI-ΔHNF4α, EnhI/II plasmids, pHBx-HA, and pMyc-TRIM21 as indicated. After 48 h post-transfection, cells were lysed, and the luciferase activity in the lysates was determined using the luciferase assay system (Promega, Madison, WI, USA) according to the manufacturer’s instructions. The signals of each sample were measured in a luminometer.

### Southern blot

The replication of HBV was analyzed by Southern blot analysis as described in our previous report with some modifications (26). Cells were harvested at 72 h post-transfection and lysed with HEPES lysis buffer containing 1% NP-40. To remove transfected plasmids, the lysates were treated with DNase I (Roche, Mannheim, Germany) at 37°C for 2 h. HBV capsids were precipitated with polyethylene glycol solution on ice overnight. After incubation, HBV capsids were digested with 20 mg/mL proteinase K (Roche, Mannheim, Germany) at 37°C for 3 h in the presence of 0.5% sodium dodecyl sulfate (SDS), then extracted by phenol/chloroform/isoamyl alcohol (25:24:1, Sigma-Aldrich, Saint Louis, MO, USA). Capsid-associated HBV DNA was precipitated with ethanol and 3 M sodium acetate. HBV DNA was separated on a 1% agarose gel and transferred onto a positively charged Hybond-XL membrane (GE Healthcare, Buckinghamshire, UK) by the alkaline transfer method. HBV DNA was detected with an HBV probe containing seven fragments of digoxigenin (DIG), which targets the whole genome of HBV and analyzed using Multi-Gauge Software v.3.0 (Fujifilm, Tokyo, Japan).

### Western blot

At 48 or 72 h post-transfection with the indicated plasmids, the cells were harvested and lysed with M-PER lysis buffer (Thermo Fisher, Waltham, MA, USA) containing a protease inhibitor cocktail (Roche, Mannheim, Germany). The lysates were boiled for 5 min in SDS sample buffer. The samples were separated by SDS-PAGE and transferred to a polyvinylidene fluoride (PVDF) membrane (Bio-Rad, Hercules, CA, USA). For blocking, 5% nonfat milk in Tris-buffered saline containing 0.1% Tween 20 (TBS-T) was used. The membrane was incubated with the primary antibody (as indicated in the figures) at 4°C overnight on a rocker shaker. The membrane was washed with TBS-T and incubated with a secondary antibody at room temperature for 1 h. The proteins were detected by enhanced chemiluminescence (AbClon, Seoul, Korea) using an ImageQuant 800 (Amersham, Buckinghamshire, UK).

### Northern blot

HBV RNA was determined by Northern blot as described previously (8). According to the manufacturer’s protocol, total RNA was extracted using TRI Reagent (Sigma-Aldrich, Saint Louis, MO, USA). Total RNA was separated by electrophoresis on a 1% formaldehyde-agarose gel and transferred onto a positively charged Hybond-XL membrane (GE Healthcare, Buckinghamshire, UK). To detect HBV RNA, the membrane was hybridized with the DIG probe used for Southern blot.

### Real-time quantitative PCR

cDNA was synthesized using 2 µg of total RNA and a high capacity RNA-to-cDNA kit (Applied Biosystems, Foster City, CA, USA). Real-time quantitative PCR was performed using SYBR master mix. The specific primers for HNF4α, HNF1α, HNF3β, and CCAAT/enhancer-binding protein α (C/EBPα) were described previously (8, 25). cDNA was amplified in a QuantStudio 3 Real-Time PCR instrument (Applied Biosystems, Foster City, CA, USA) under the following conditions: denaturation at 94°C for 5 min, followed by 40 cycles of 94°C for 30 s and 55°C for 1 min, and a final extension at 55°C for 5 min. Transcript levels were quantified by the comparative ΔΔCt method relative to a control sample and normalized by glyceraldehyde-3-phosphate dehydrogenase (GAPDH) (27). HBV DNA was precipitated with the protocol described for Southern blot in this study. The primers for HBV DNA were as follows: forward (nt 256 to 274), 5′-CTCGTGGTGGACTTCTCTC-3′; and reverse (nt 404 to 421), 5′-CTGCAGGATGAAGAGGAA-3′. Total HBV DNA was amplified in a QuantStudio 3 Real-Time PCR instrument (Applied Biosystems, Foster City, CA, USA) under the following conditions: denaturation at 94°C for 5 min, followed by 40 cycles of 94°C for 30 s and 55°C for 1 min, and a final extension at 55°C for 5 min. Serial dilutions of pHBV1.2(+) were used as a quantification standard.

### Enzyme-linked immunosorbent assay (ELISA)

Culture supernatants were collected at 72 h post-transfection. The levels of secreted HBeAg and HBsAg were measured by ELISA using a kit (Wantai Pharm Inc., Beijing, China) in accordance with the manufacturer’s instructions. The optical density (OD) values were measured at a wavelength of 450 nm using a spectrophotometer (SpectraMAX Plus 384).

### Co-immunoprecipitation

At 48 h post-transfection, cells were harvested and lysed by Pierce IP lysis buffer (Thermo Fisher Scientific, Waltham, MA, USA) with a protease inhibitor cocktail (Roche, Mannheim, Germany). For input, 20% of lysates were mixed with SDS sample buffer and boiled for 5 min. The remaining lysates were pre-cleaned by A-agarose Bead (Roche, Mannheim, Germany) at 4°C for 2 h. The clarified lysates were incubated with the primary antibody on an orbital shaker at 4°C overnight. The antibody-protein complexes were precipitated with A-agarose Bead at 4°C for 4 h, washed three times with cold phosphate-buffered saline (PBS), and boiled for 5 min with SDS sample buffer. The samples were analyzed by Western blot.

### Ubiquitination assay

HA-tagged ubiquitin plasmids were co-transfected with the pMyc vector or pMyc-TRIM21. Forty-three hours after co-transfection, MG132 (20 µM) was treated for 5 h. The cells were lysed by SDS lysis buffer and boiled for 10 min. For the input control, 10% of lysates were mixed with SDS sample buffer and analyzed by Western blot. The remaining lysates were diluted 1:5 with TBS containing a protease inhibitor cocktail and incubated with the anti-HA antibody at 4°C overnight. The poly-ubiquitinated proteins were precipitated with protein A-agarose and were detected by Western blot.

### Generation of TRIM21 knock-out cell line

To produce lentivirus, pCMV delta R8.2 and pCMV-VSV-G were co-transfected with pLentiCRISPR V2-TRIM21 (ratio; 1:1:1) into the HEK293T cell line by Lipofectamine 2000. At 48 h post-transfection, the supernatant was collected and filtered through a 0.45 µm pore filter. The medium containing lentivirus was mixed with fresh medium supplemented with polybrene (12 µg/ml) in a 1:1 ratio and treated to HepG2 cell line. Infected cells were selected by puromycin (Sigma-Aldrich, Saint Louis, MO, USA). To confirm TRIM21 knock-out cells, the endogenous TRIM21 protein level was analyzed by Western blot.

### HBV production and infection

To obtain HBV virions, as described previously, culture supernatants of HepAD38 cells in the absence of tetracycline were collected, centrifuged, and filtered with a 0.45 µm pore size filter. To precipitate HBV particles, the filtered supernatant was mixed with 6% PEG8000 (Sigma-Aldrich, Saint Louis, MO, USA) and incubated at 4°C overnight. The mixtures were centrifuged, and a pellet containing HBV virions was re-suspended with PBS with 25% FBS. Cells were infected with 1,000 genome equivalents per cell (Geq/cell) in fresh primary hepatocyte maintenance medium (PMM) containing 4% PEG. At 16 h post-infection, the cells were washed three times with PBS and maintained in fresh PMM containing 2.5% dimethyl sulfoxide (DMSO).

### Isolation of PHHs

Human liver tissue specimens proven negative for HAV, HBV, HCV, and HDV were obtained by therapeutic hepatectomies. PHHs were isolated from the human liver by using a two-step collagenase perfusion, as explained in our previous report (28). The liver tissue was perfused through blood vessels on the surface with warm perfusion buffer containing collagenase (0.5 g/L, Sigma-Aldrich, Saint Louis, MO, USA) and calcium chloride (0.56 g/L, Sigma). The isolated PHHs were filtered with stainless steel meshes in two steps (pore size, 300 and 150 µm) and seeded on collagen-coated plates (Corning, Tewksbury, MA, USA).

### Hydrodynamic injection

C57BL/6 mice (male, 6 weeks old) were hydrodynamically co-injected with pHBV1.2(+) or pHBV1.2(−) andpMyc-TRIM21 plasmids in PBS. The volume equivalent to 10% of the mouse body weight was injected via the intravenous tail vein with high pressure within 4–6 s. To analyze HBV replication, the mice were sacrificed 96 h post-injection.

### Statistical analysis

At least three independent experiments were performed for all analyses. Data are presented as mean ± SD. Statistical significance was evaluated by one-way analysis of variance or Student’s *t*-test in GraphPad Prism v.6.

## RESULTS

### Cytokine-inducible TRIM21 down-regulates HBV replication

**Fig 1:**
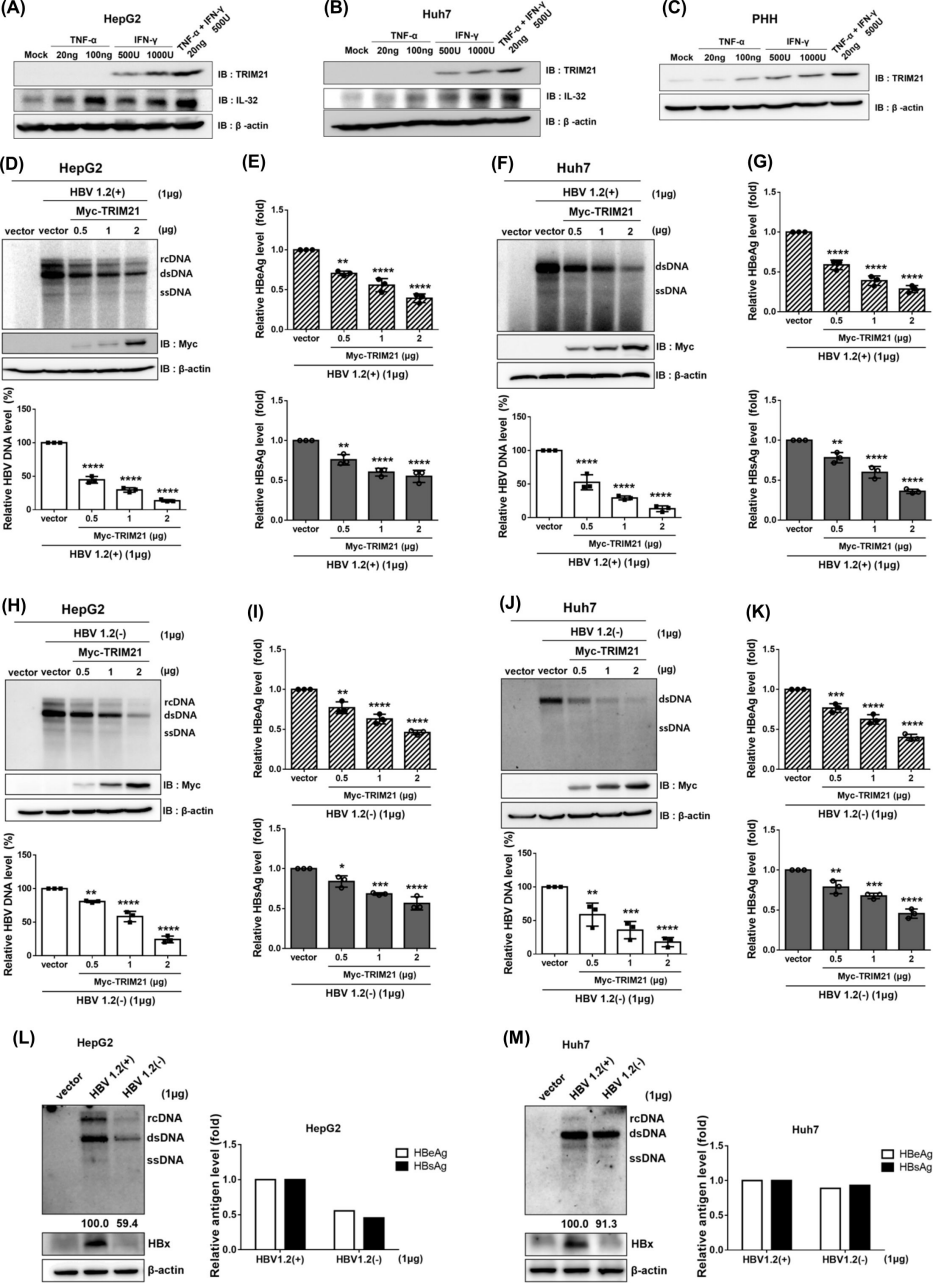
Cytokine-inducible TRIM21 inhibits HBV replication. Induction of TRIM21 by cytokines in HepG2 (**A**), Huh7 (**B**), and PHHs (**C**). Cells were treated with IFN-γ and/or TNF-α. At 48 h post-treatment, the cells were lysed and analyzed by Western blot. IL-32 was used as a positive control for cytokine-induced response (**A–C**). (**D–K**) HepG2 cells and Huh7 cells were co-transfected with the indicated plasmids. At 72 h post-transfection, cells and supernatants in cell culture were harvested to measure HBV DNA, HBeAg, and HBsAg. (**D and H**) Cellular HBV DNA in pHBV1.2(+) or pHBV1.2(−) and pMyc-TRIM21-transfected HepG2 were analyzed by Southern blot, and HBeAg and HBsAg were measured using ELISA (**E and I**). For panels F and J, HBV replication for Huh7 was determined by Southern blot; for panels G and K, relative HBV antigen levels were measured by ELISA. Expression of Myc-TRIM21 protein was detected by Western blot with Myc antibody in all experiments. (**L and M**) Comparison of HBV replication levels between pHBV1.2(+) and pHBV1.2(−) plasmids by Southern blot and ELISA, and confirmation of HBx protein expression by Western blot in HepG2 (**L**) and Huh7 (**M**). HBV replication was quantified using Multi-Gauge v.3.0 and plotted. Data were obtained from at least three independent experiments (mean ± SD) (**D–K**). *, *P* < 0.05; **, *P* < 0.01; ***, *P* < 0.001; ****, *P* < 0.0001.

In a previous study, various cytokines were found to regulate HBV gene expression and replication through diverse mechanisms (29, 30). TNF-α and IFN-γ have been shown to suppress HBV in a non-cytolytic manner (31, 32). TRIM21 has been reported as an interferon-inducible E3-ubiquitin ligase protein (33). In a previous study, it was revealed that the interferon-stimulated response element (ISRE) motif exists in the promoter of TRIM21, and its expression is regulated by IFN regulatory factors (34). We investigated whether the induction of TRIM21 occurs in response to TNF-α and IFN-γ in human hepatoma cell lines and PHHs. Cells were treated with TNF-α and IFN-γ at the concentrations indicated in the figure for 48 h. In hepatoma cell lines HepG2 and Huh7, TRIM21 was significantly increased by IFN-γ but not TNF-α. Notably, there was no change in TRIM21 levels with TNF-α treatment alone, but combined treatment with IFN-γ showed a synergistic induction of TRIM21 (Fig. 1A and B). IFN-γ remarkably increased TRIM21 in PHHs, and unlike in the hepatoma cell lines, TRIM21 levels were also increased by TNF-α. The combination of TNF-α and IFN-γ resulted in a synergistic increase in TRIM21 levels in PHHs as well (Fig. 1C). These data confirmed that TRIM21 can be induced by cytokines in hepatoma cell lines and PHHs.

To investigate the antiviral effect of TRIM21 against HBV, capsid-associated HBV DNA, HBeAg, and HBsAg levels were measured. HepG2 and Huh7 cells were co-transfected with a TRIM21-expressing plasmid and the replication-competent HBV plasmid HBV1.2(+). HBV replication was dramatically decreased by TRIM21 in a dose-dependent manner (Fig. 1D), along with the expression of HBeAg (Fig. 1E, upper panel) and HBsAg (Fig. 1E, bottom panel) in HepG2. In the other human hepatoma cell line Huh7, TRIM21 repressed HBV replication (Fig. 1F), HBeAg (Fig. 1G, upper panel), and HBsAg levels (Fig. 1G, bottom panel) similarly to HepG2.

In a previous study, TRIM21 targeted HBx and caused proteasomal degradation to restrict HBV (24). Interestingly, we found that TRIM21 still strongly inhibits HBV replication even when an HBx-deficient HBV plasmid HBV1.2(−) was used. In HepG2 cells, although the replication level was low, TRIM21 reduced HBV replication (Fig. 1H) as well as HBeAg (Fig. 1I, upper panel) and HBsAg (Fig. 1I, bottom panel) in the absence of HBx. Consistently, in Huh7 cells, TRIM21 demonstrated antiviral effect on HBV replication and secreted antigens in the absence of HBx (Fig. 1J and K). HBV1.2(+) and HBV1.2(−) plasmids were transfected into HepG2 (Fig. 1L) and Huh7 cells (Fig. 1M) to compare HBV replication and HBx expression levels. The obtained data further suggest that in addition to reducing HBx, TRIM21 may inhibit HBV replication through another mechanism.

### Deficiency of TRIM21 restored HBV replication suppressed by cytokine treatment

**Fig 2:**
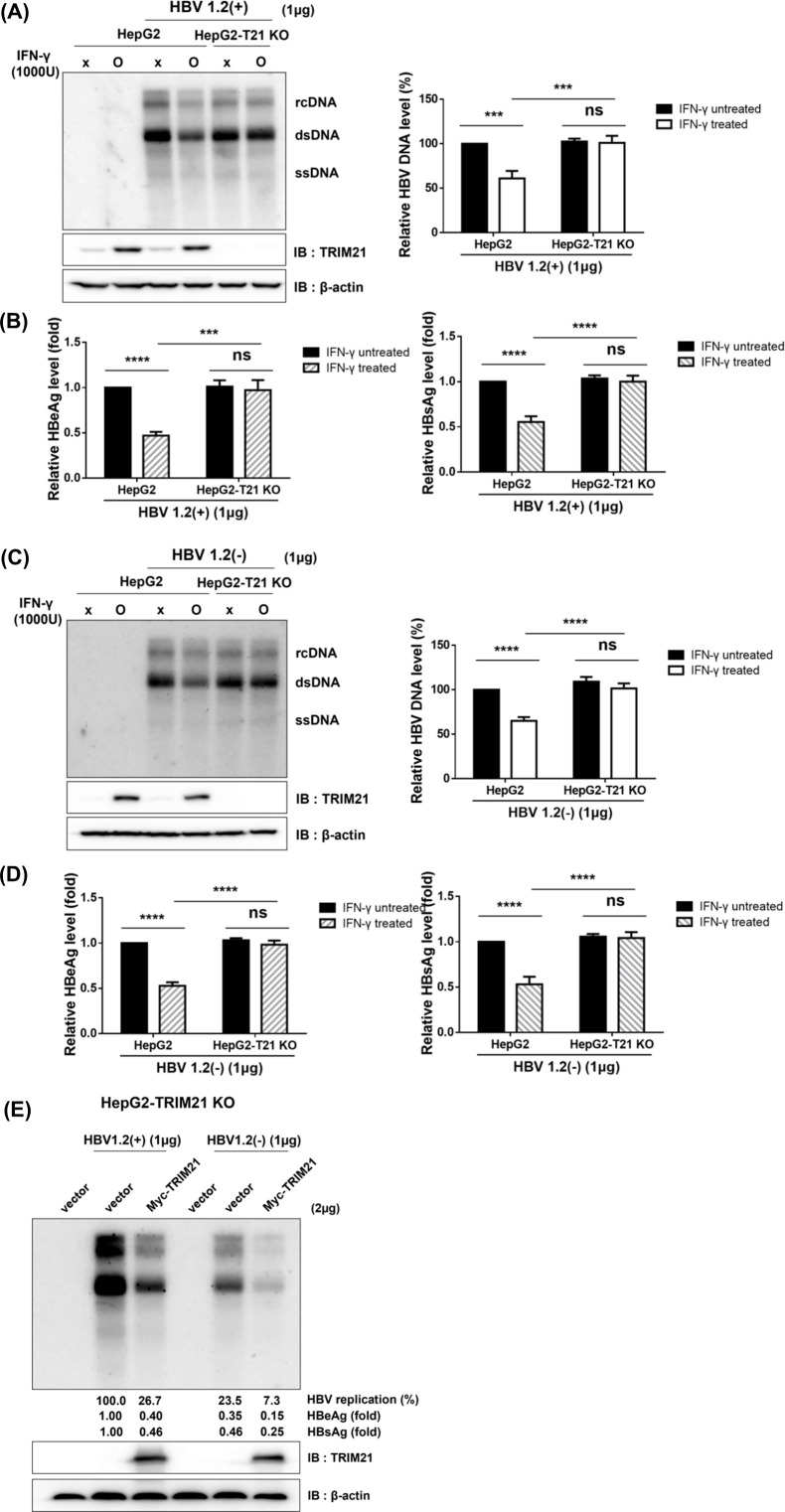
Cytokine-mediated repression of HBV replication was restored in TRIM21 knock-out cells. (**A–D**) Cells were transfected with pHBV1.2(+) or pHBV1.2(−). At 24 h post-transfection, IFN-γ (1,000 U) was treated three times at 24-h intervals, then cells were harvested and analyzed at 72 h post-transfection. (**A**) Southern blot and Western blot analysis for HBV replication and TRIM21 expression from pHBV1.2(+) transfected and IFN-γ treated or non-treated cells, and (**B**) HBeAg and HBsAg levels in supernatants of cell culture were measured using ELISA. (**C**) Relative HBV replication level in pHBV1.2(−) transfected cells treated with or without IFN-γ was determined by Southern blot, and (**D**) levels of HBeAg and HBsAg were analyzed by ELISA. Expression of TRIM21 protein was detected by immunoblot with TRIM21 antibody in both experiments. (**E**) HepG2-T21 KO cells were co-transfected with pHBV1.2(+) or pHBV1.2(−) and pMyc-TRIM21. Seventy-two hours post-transfection, cells were lysed, and HBV replication was analyzed by Southern blot and ELISA. TRIM21 protein was detected by Western blot. Replication of HBV was quantified using Multi-Gauge v.3.0 and plotted. At least three independent experiments were performed. Data are means ± the SD. ns, not significant; *, *P* < 0.05; **, *P* < 0.01; ***, *P* < 0.001; ****, *P* < 0.0001.

To determine whether cytokine-induced TRIM21 participates in the cytokine-mediated antiviral effect against HBV, we generated TRIM21 knock-out cells from HepG2 cells (HepG2-T21 KO). TRIM21 knock-out cells were transfected with pHBV1.2(+) and pHBV1.2(−) , and IFN-γ was administered. In the absence of TRIM21, IFN-γ could not inhibit the replication of HBV in HepG2 cells (Fig. 2A). Moreover, the deficiency of TRIM21 resulted in the failure of down-regulation of HBeAg (Fig. 2B, left panel) and HBsAg (Fig. 2B, right panel). The absence of TRIM21 negated the cytokine-induced decrease in HBV replication, even when HBx was not present (Fig. 2C). Additionally, cytokine treatment could not suppress HBeAg (Fig. 2D, left panel) and HBsAg levels (Fig. 2D, right panel) in the absence of HBx in the HepG2-T21 KO cells (Fig. 2D). Moreover, ectopic expression of TRIM21 effectively inhibited HBV replication in the presence or absence of HBx (Fig. 2E). These results indicate that TRIM21 plays a key role in cytokine-induced anti-HBV effect.

### TRIM21 inhibits HBV transcription through regulating the activity of HBV enhancers

**Fig 3:**
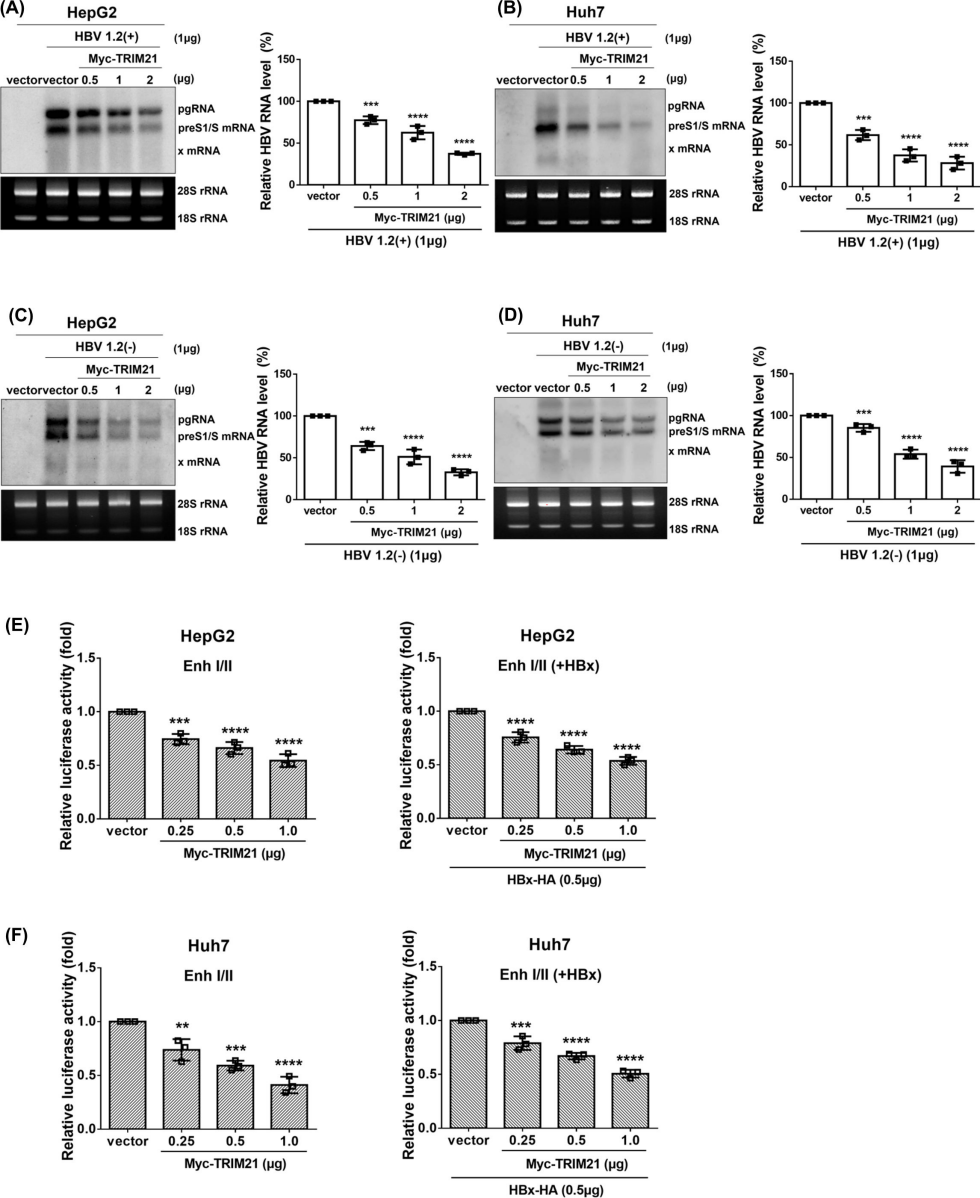
Overexpression of TRIM21 regulates HBV transcription. (**A–D**) To determine HBV transcription level, cells were transfected with plasmids indicated in the figure. At 72 h post-transfection, cells were harvested and analyzed. (**A and C**) HBV RNAs were determined by Northern blot in pHBV1.2(+) or pHBV1.2(-), and pMyc-TRIM21 transfected HepG2 cells. (**B and D**) HBV RNAs were analyzed by Northern blot in Huh7 cells. HBV RNAs were quantified using Multi-Gauge v.3.0 and plotted. (**E–F**) Effect of TRIM21 on the activity of HBV enhancers I and II in the presence or absence of HBx. Cells were co-transfected with plasmids for EnhI/II-luc, β-gal, pGL3-basic, pHBx-HA, and pMyc-TRIM21 as indicated. At 48 h post-transfection, relative luciferase activity was analyzed by luciferase assay in HepG2 (**E**) and Huh7 (**F**) cells. Data are means ± the SD. **, *P* < 0.01; ***, *P* < 0.001; ****, *P* < 0.0001. At least three independent experiments were performed.

The above data demonstrate that the overexpression of TRIM21 decreases HBV replication. To determine at what stage TRIM21 works, we next investigated whether it regulates HBV transcription. In HepG2 cells, Northern blot analysis showed that all HBV transcripts were significantly decreased by TRIM21 in a dose-dependent manner (Fig. 3A). TRIM21 also decreased HBV transcription in Huh7 cells to a similar extent as in HepG2 cells (Fig. 3B). To exclude the effect of HBx, cells co-transfected with pHBV1.2(−) and pMyc-TRIM21 were analyzed. HBV transcripts were efficiently decreased by TRIM21 when HBx was absent in HepG2 cells (Fig. 3C). TRIM21 down-regulated HBV transcription in Huh7 cells in the absence of HBx (Fig. 3D). Since the transcription of HBV is controlled by two essential enhancers (EnhI, EnhII) (30), we used a luciferase reporter plasmid containing EnhI and EnhII and analyzed the regulation of luciferase activity by TRIM21. Activities of enhancers were down-regulated by TRIM21 in the presence or absence of HBx in HepG2 cells (Fig. 3E). The same results were obtained in Huh7 cells (Fig. 3F). These data suggest that TRIM21 inhibits HBV at the transcriptional level through the down-regulation of HBV enhancer I and II activities.

### RING and PRY-SPRY domains are required for antiviral activity of TRIM21 against HBV

**Fig 4:**
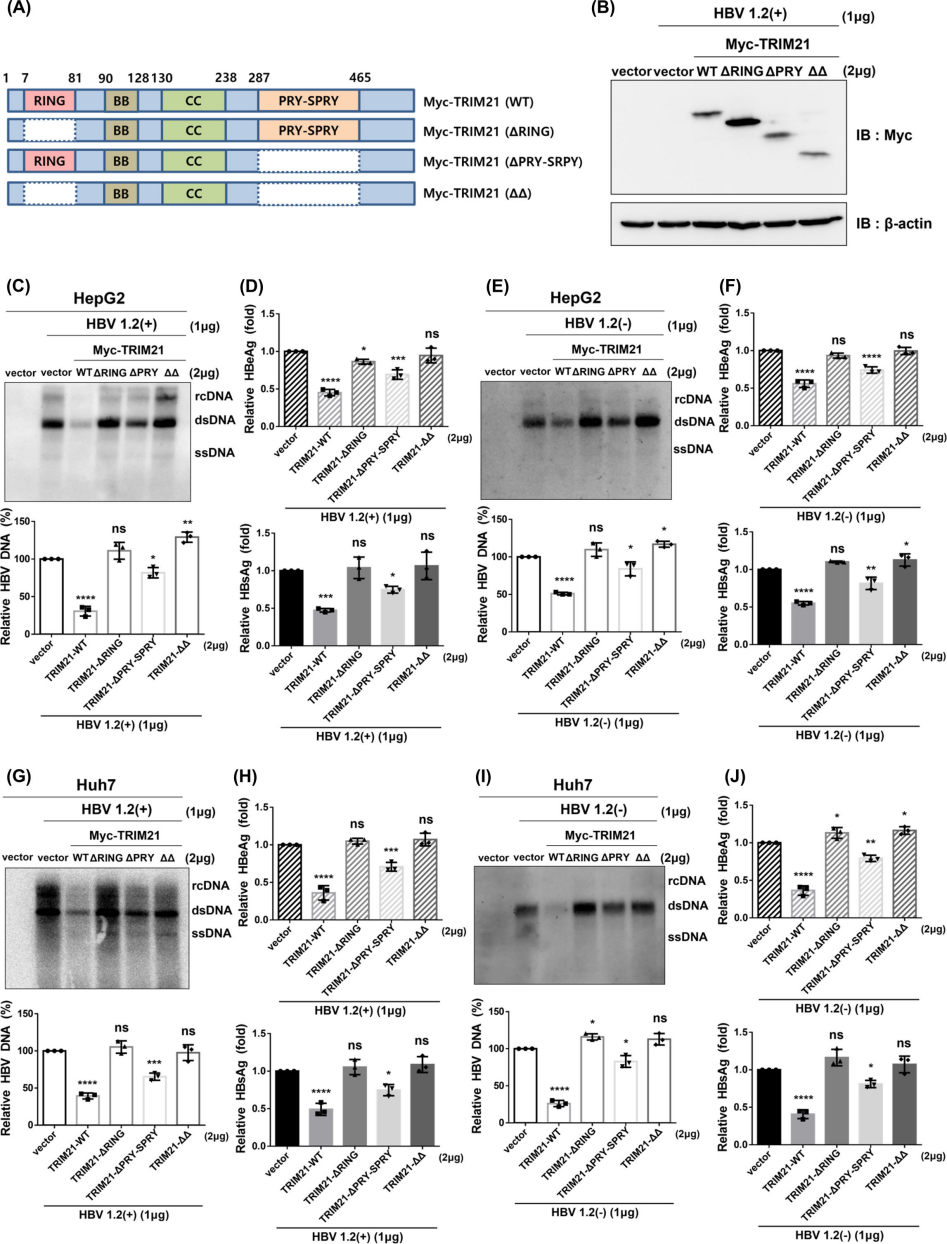
RING and PRY-SPRY domains of TRIM21 participate in antiviral activity against HBV. (**A**) A schematic diagram of the TRIM21 deletion mutants. (**B–F**) HepG2 cells were co-transfected with pHBV1.2(+) or pHBV1.2(−) and TRIM21 mutant plasmids. At 72 h post-transfection, cells were harvested and analyzed. (**B**) Expression of protein for each plasmid was detected by immunoblot with Myc antibody. (**C and E**) HBV DNA level was analyzed by Southern blot. (**D and F**) HBeAg and HBsAg in supernatants of cell culture were measured using ELISA. (**G and J**) Huh7 cells were co-transfected with pHBV1.2(+) or pHBV1.2(−) and TRIM21 mutant plasmids. Cells were harvested and examined at 72 h post-transfection. (**G and I**) HBV DNA level was determined by Southern blot. (**H and J**) Relative HBsAg and HBeAg levels in the supernatant of cell culture were measured by ELISA. HBV DNA was quantified using Multi-Gauge v.3.0 and plotted. Data are means ± the SD. ns, not significant; *, *P* < 0.05; **, *P* < 0.01; ***, *P* < 0.001; ****, *P* < 0.0001. At least three independent experiments were performed.

TRIM21 comprises RING, B-Box, coiled-coil domains, and PRY-SPRY domain. To identify which domain of TRIM21 participates in the anti-HBV effect, we designed and constructed various deletion mutants of TRIM21 (Fig. 4A). Each construct was transfected into HepG2 cells, and protein expression was confirmed by Western blot (Fig. 4B). TRIM21 wild-type significantly reduced HBV DNA, HBeAg, and HBsAg as expected, but the RING domain deletion (ΔRING) and RING/PRY-SPRY deletion mutant (ΔΔ) lost the antiviral effect in the presence of HBx (Fig. 4C and D) or in its absence (Fig. 4E and F) in HepG2 cells. Also, the PRY-SPRY deletion mutant (ΔPRY-SPRY) slightly abolished the anti-HBV effect in the presence of HBx (Fig. 4C and D) or not (Fig. 4E and F). We obtained consistent results in Huh7 cells as in HepG2 cells (Fig. 4G to J). These results suggest that the RING and PRY-SPRY domains of TRIM21 are required for antiviral activity against HBV.

### TRIM21 regulates expression of HNFs

**Fig 5:**
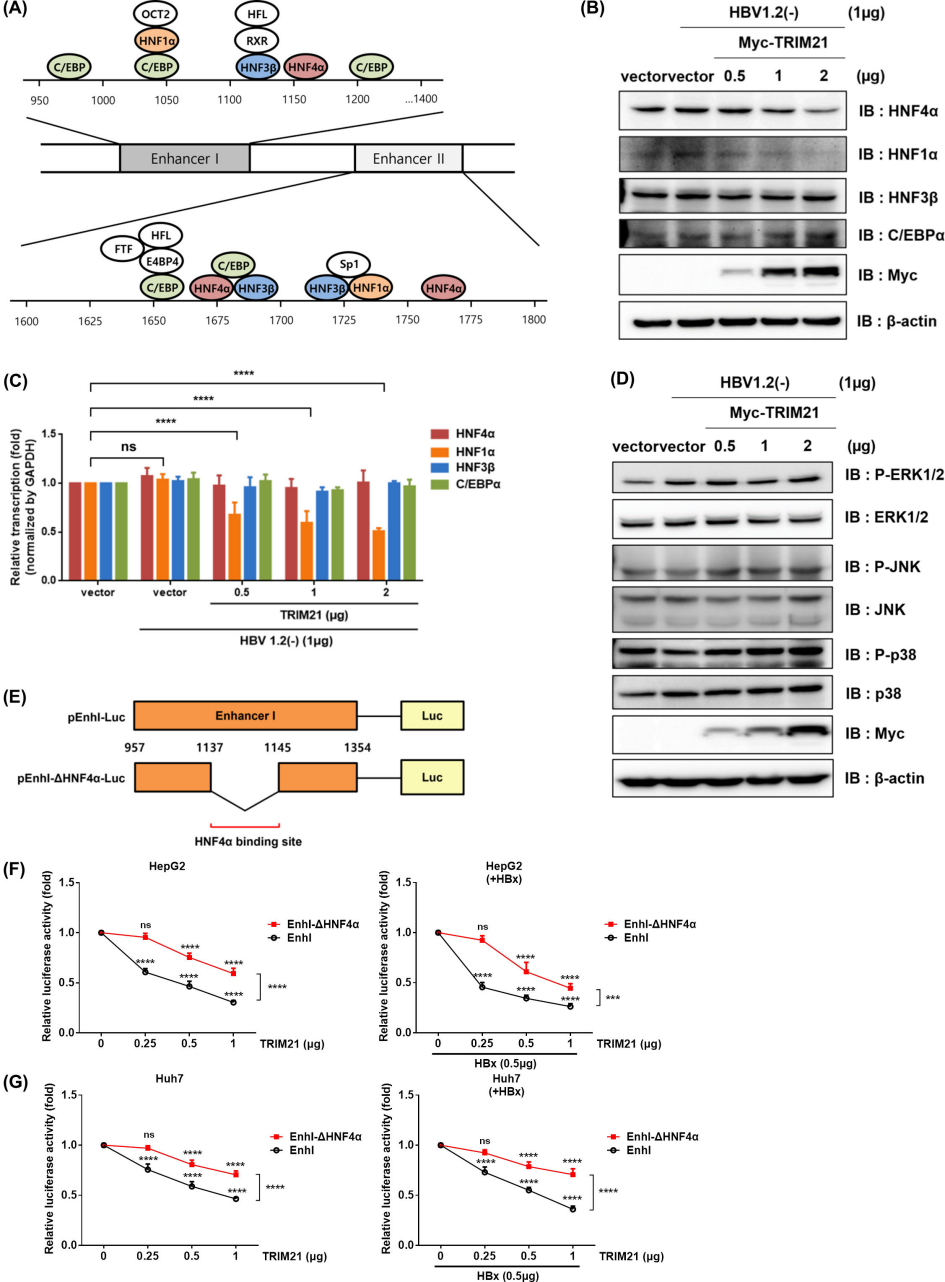
TRIM21 down-regulates expression of HNF4α and HNF1α. (**A**) Schema of liver-enriched transcription factors associating with HBV enhancers. (**B–D**) Effect of TRIM21 on the expression of HNFs. HepG2 cells were co-transfected with pHBV1.2(−) and pMyc-TRIM21. At 48 h post-transfection, the expression level of each protein or mRNA indicated in the figure was analyzed by Western blot (**B**) and real-time PCR (**C**). The relative transcription level of each gene was normalized by the GAPDH gene. Data are means ± the SD. ****, *P* < 0.0001. (**D**) Activation of MAPK signaling pathways was analyzed by immunoblotting. (**E**) Demonstration of EnhI and EnhI-ΔHNF4α luciferase reporter plasmid constructs. (**F and G**) Comparison of the effects of TRIM21 on pEnhI-luc and pEnhI-ΔHNF4α-luc, pβ-gal, pHBx-HA, and pMyc-TRIM21. At 48 h post-transfection, relative luciferase activity was analyzed by luciferase reporter assay. At least three independent experiments were performed.

The preceding data indicate that TRIM21 reduces the activity of both EnhI and EnhII. We hypothesized that TRIM21 regulates transcription factors that bind to enhancers. The liver-enriched transcription factors, including HNF4α, HNF1α, HNF3β, and C/EBPα, which are key transcription factors for cccDNA transcription, have been known to bind EnhI and EnhII (Fig. 5A) (4). To determine whether TRIM21 regulates these transcription factors and to show that HBx is not involved, pHBV1.2(−) was co-transfected with pMyc-TRIM21 into HepG2 cells, and protein level and mRNA level were analyzed by Western blot and qRT-PCR, respectively. At the protein level, overexpression of TRIM21 decreased HNF1α and HNF4α, activators for HBV enhancers, in the absence of HBx, but HNF3β and C/EBPα levels were not changed (Fig. 5B). The mRNA level of HNF1α was down-regulated by TRIM21, but there was no significant change for HNF4α, HNF3β, and C/EBPα (Fig. 5C). Interestingly, these data showed that TRIM21 can suppress the transcription of HNF1α but not HNF4α, and that TRIM21 regulates HNF4α post-translationally. As the MAPK signaling pathway regulated the expression of HNFs in our previous study (8), we checked whether TRIM21 can control the expression of factors in this pathway. HepG2 cells were co-transfected with pHBV1.2(−) and pMyc-TRIM21 to analyze each protein by Western blot. In the MAPK pathway, there was no meaningful activation or change in any factors (Fig. 5D). To confirm whether the decrease in HBV enhancer activity was due to the TRIM21-mediated reduction of HNF4α, an enhancer I mutant with a deletion of the HNF4α binding site (nt1138-1144, gaaccttta) (28, 35) was generated (Fig. 5E). When the HNF4α binding site was interrupted, the decrease in enhancer activity by TRIM21 was alleviated in the presence or absence of HBx in HepG2 (Fig. 5F) and Huh7 (Fig. 5G) cells. These results suggest that TRIM21 regulates HNF1α at the transcriptional level and HNF4α at the post-transcriptional level, and not through the MAPK signaling pathway.

### TRIM21 promotes proteasomal degradation of HNF4α through poly-ubiquitination

**Fig 6:**
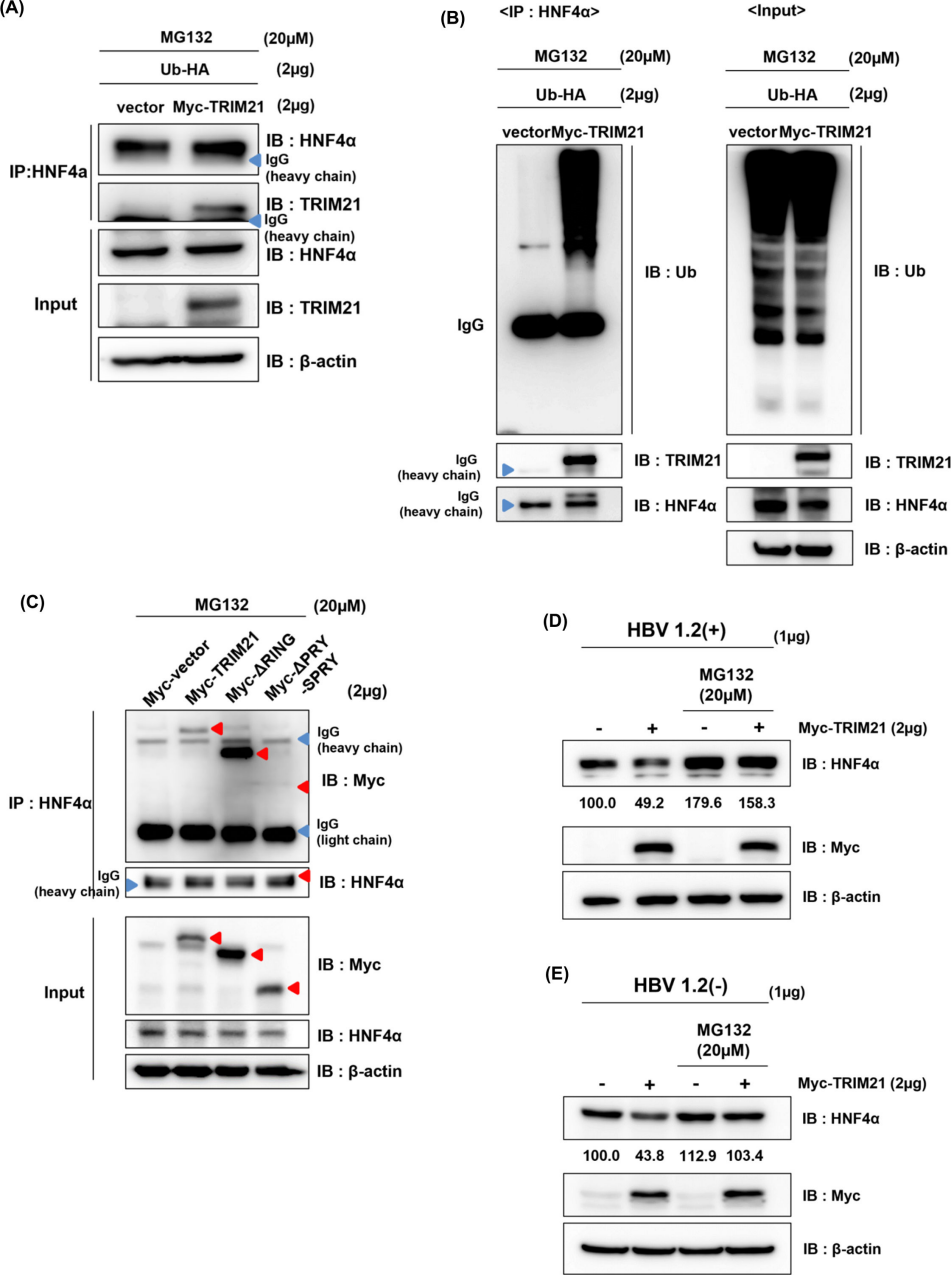
TRIM21 promotes the ubiquitin-dependent degradation of HNF4α. (**A and B**) HepG2 cells were co-transfected with pUb-HA and pMyc-TRIM21. At 42 h post-transfection, cells were treated with MG132 (20 µM) for 5 h and subjected to co-immunoprecipitation with HNF4α antibody. (**A**) Proteins in the immunoprecipitated complex were analyzed by Western blot with antibodies indicated in the figure. (**B**) The level of poly-ubiquitinated HNF4α was determined using immunoblot with Ub antibody. (**C**) pMyc-TRIM21, pMyc-ΔRING, or pMyc-ΔPRY-SPRY constructs were transfected into the HepG2 cells and interaction with HNF4α analyzed by co-immunoprecipitation. (**E**) Recovery of HNF4α protein with a proteasome inhibitor. pHBV1.2(+) or pHBV1.2(−) and pMyc-TRIM21 were co-transfected into HepG2 cells. At 42 h post-transfection, cells were treated with MG132 (20 µM) before harvesting. The level of HNF4α protein was determined by Western blot.

As HNF4α was down-regulated by TRIM21 in post-transcriptional regulation, and the RING domain, which has E3 ligase activity, was involved in the inhibition of HBV replication in the above data, we investigated whether TRIM21 interacts with HNF4α and promotes poly-ubiquitination for degradation. HepG2 cells were co-transfected with pMyc-TRIM21 and pUb-HA, and MG132 was treated for 5 h before harvest. Protein complexes were immunoprecipitated with HNF4α antibody, and found that TRIM21 interacts with HNF4α, as detected by immunoblot (Fig. 6A). The ubiquitination assay showed that TRIM21 significantly increased poly-ubiquitination of HNF4α (Fig. 6B, left panel, lane 2). To find out which domain is responsible for TRIM21 and HNF4α interaction, wild-type Myc-TRIM21, Myc-TRIM21 RING domain deletion mutant (Myc-ΔRING), and Myc-TRIM21 PRY-SPRY deletion mutant (Myc-ΔPRY-SPRY) plasmids were transfected into HepG2 cell and samples were subjected to co-immunoprecipitation. Interestingly, Myc-TRIM21 and Myc-ΔRING could bind to HNF4α, while Myc-ΔPRY-SPRY failed to interact with HNF4α, meaning that the PRY-SPRY domain of TRIM21 is crucial for its interaction with HNF4α (Fig. 6C). When MG132, a proteasome inhibitor, was treated, the decrease of HNF4α protein by TRIM21 was recovered in the presence of HBx (Fig. 6D) or not (Fig. 6E). These data demonstrate that TRIM21 promotes the degradation of HNF4α through the ubiquitin-proteasome system.

### TRIM21 regulates HBV replication *in vivo*

**Fig 7:**
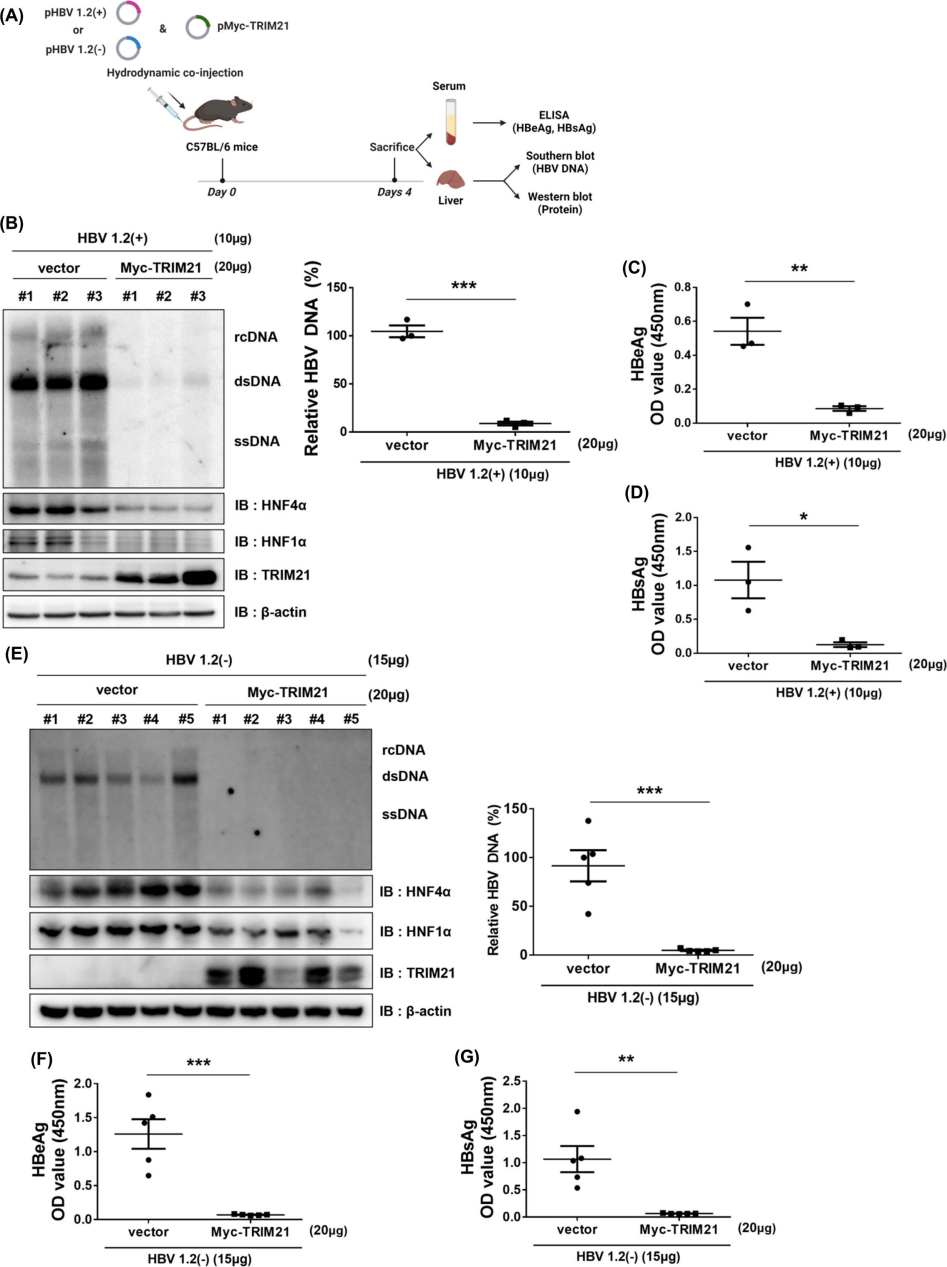
TRIM21 inhibits HBV in a hydrodynamic injection mouse model. (**A**) The scheme of the *in vivo* test with hydrodynamically injected C57BL/6 mice (male, 6 weeks old). Mice were hydrodynamically co-injected with the plasmids indicated in the figure. At 4 days post-injection, mice were sacrificed and analyzed. (**B and E**) Suppression of HBV replication by TRIM21 *in vivo*. HBV DNA level in pHBV1.2(+) (**B**) or pHBV1.2(−) (**E**) and pMyc-TRIM21 co-injected mouse liver tissue was determined by Southern blot. HBV DNA level was quantified using Multi-Gauge v.3.0 and plotted. HNF4α, HNF1α, and TRIM21 proteins in mouse liver tissue were detected by Western blot. (**C,D, F, and G**) HBV antigen measurements in mice co-injected with pHBV1.2(+) (**C and D**) or pHBV1.2(−) (**F and G**) and pMyc-TRIM21 mouse. HBeAg and HBsAg levels in the serum of mice were measured by ELISA. Data are means ± the SD. The statistical significance of the differences was assessed by the Student’s *t*-test: *, *P* < 0.05; **, *P* < 0.01; ***, *P* < 0.001.

To confirm the anti-HBV activity of TRIM21 *in vivo*, C57BL/6 mice were hydrodynamically co-injected with pHBV1.2(+) (Fig. 7B through D) or pHBV1.2(−) (Fig. 7E through G) and pMyc-TRIM21. Mice were sacrificed at 4 days post-injection, and HBeAg and HBsAg in the serum and HBV DNA in the liver tissue were analyzed by ELISA and Southern blot, respectively (Fig. 7A). TRIM21 dramatically suppressed the replication of HBV in mouse livers (Fig. 7B and E). Consistent with the results *in vitro*, HNF4α and HNF1α protein levels were strongly decreased by the overexpression of TRIM21 in mouse livers (Fig. 7B and E). HBeAg (Fig. 7C and F) and HBsAg (Fig. 7D and G) were also significantly decreased by TRIM21. These data demonstrate that TRIM21 efficiently inhibits HBV replication *in vivo*, presumably by reducing HNFs.

### TRIM21 inhibits replication of HBV in PHHs

**Fig 8:**
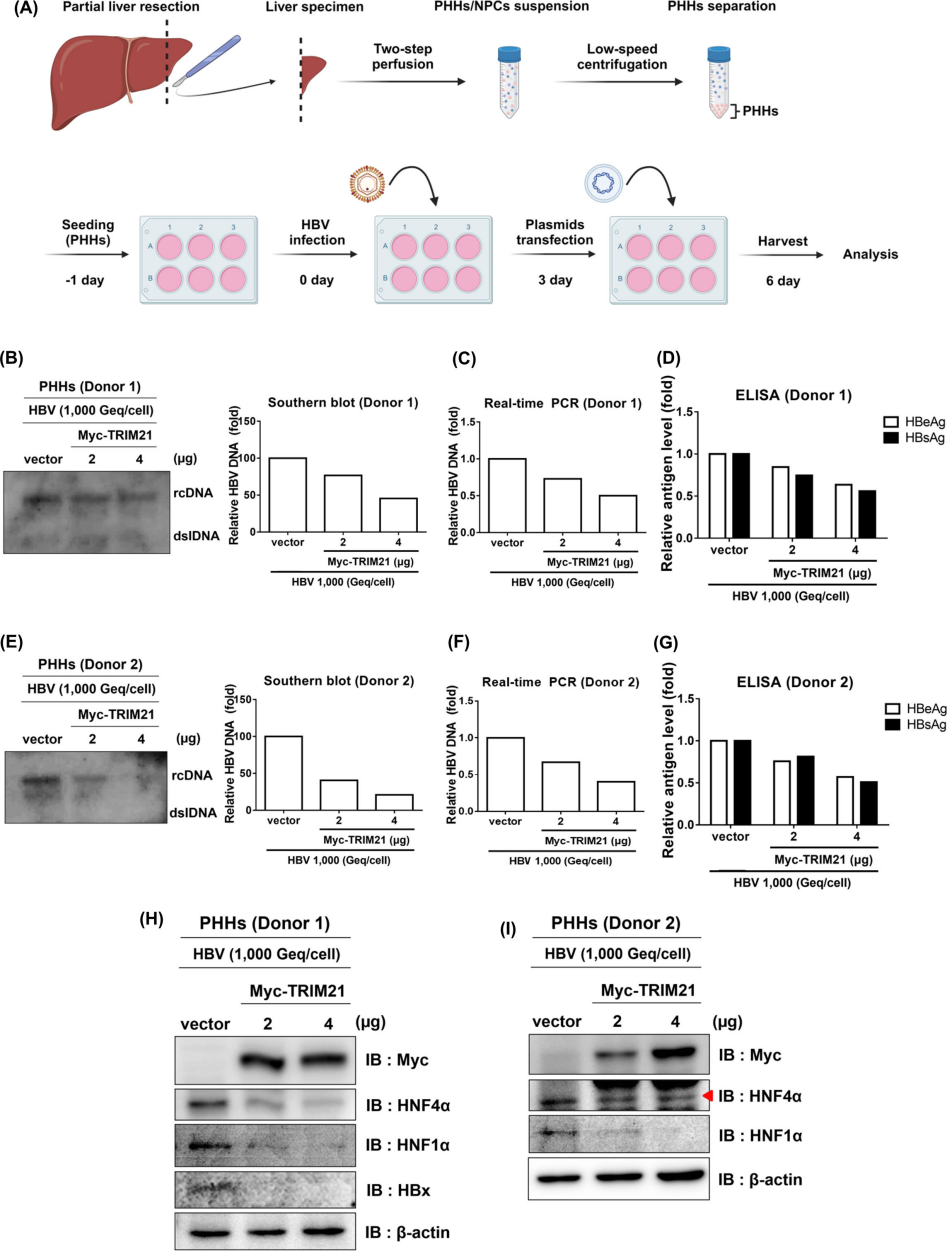
TRIM21 suppresses viral replication in HBV-infected PHHs. (**A**) The workflow of the analysis for HBV-infected PHHs. Liver specimens were resected from a patient. PHHs/non-parenchymal cells (NPCs) suspension was obtained by a two-step perfusion from liver specimens. PHHs were separated with low-speed centrifugation and seeded on a collagen-coated six-well plate. The day after seeding, PHHs were infected with HBV virions (1,000 Geq/cell). At 3 days post-infection, pMyc vector or pMyc-TRIM21 was transfected into PHHs. Six days after infection, cells and supernatants of cell culture were harvested and analyzed. (**B–D**) Effect of TRIM21 overexpression on HBV replication in PHHs (donor 1). HBV DNA level was determined by Southern blot (**B**) and real-time PCR (**C**). (**D**) HBV antigens in PHHs (donor 1) were analyzed using ELISA. (**E–G**) Reduction of HBV replication by TRIM21 in PHHs (donor 2). HBV replication was analyzed by Southern blot (**E**) and real-time PCR (**F**). HBeAg and HBsAg levels were measured by ELISA (G). HBV replication was quantified using Multi-Gauge v.3.0 and plotted. (**H and I**) Regulation of HNF4α and HNF1α by TRIM21 in PHHs. Expression levels of HNF4α, HNF1α, and Myc-TRIM21 proteins in both PHHs (donor 1, **H**; donor 2, **I**) were determined by immunoblot.

To show the relevance of human HBV infection, PHHs obtained from patients’ liver tissues were used to confirm the anti-HBV effect of TRIM21. PHHs were obtained from the liver tissues following the workflow described in Fig. 8A, and infected with HBV generated from HepAD38 cells. Subsequently, infected PHHs were transfected with a plasmid expressing TRIM21 and analyzed (Fig. 8A). Replication of HBV was analyzed by Southern blot (Fig. 8B) and quantitative real-time PCR (Fig. 8C). TRIM21, although not easily detectable by Southern blot, inhibited clearly HBV replication in a dose-dependent manner in PHHs. HBeAg and HBsAg levels were decreased by the overexpression of TRIM21 (Fig. 8D). For PHHs (donor 2), TRIM21 clearly showed an anti-HBV effect similar to the results in donor 1 (Fig 8E and G). The expression of HNF4α and HNF1α proteins was suppressed by TRIM21 overexpression in both PHHs from two donors (Fig. 8H and I). These data collectively indicate that TRIM21 plays a role in the inhibition of HBV replication through decreasing the expression of HNF4α and HNF1α in PHHs.

## DISCUSSION

**Fig 9:**
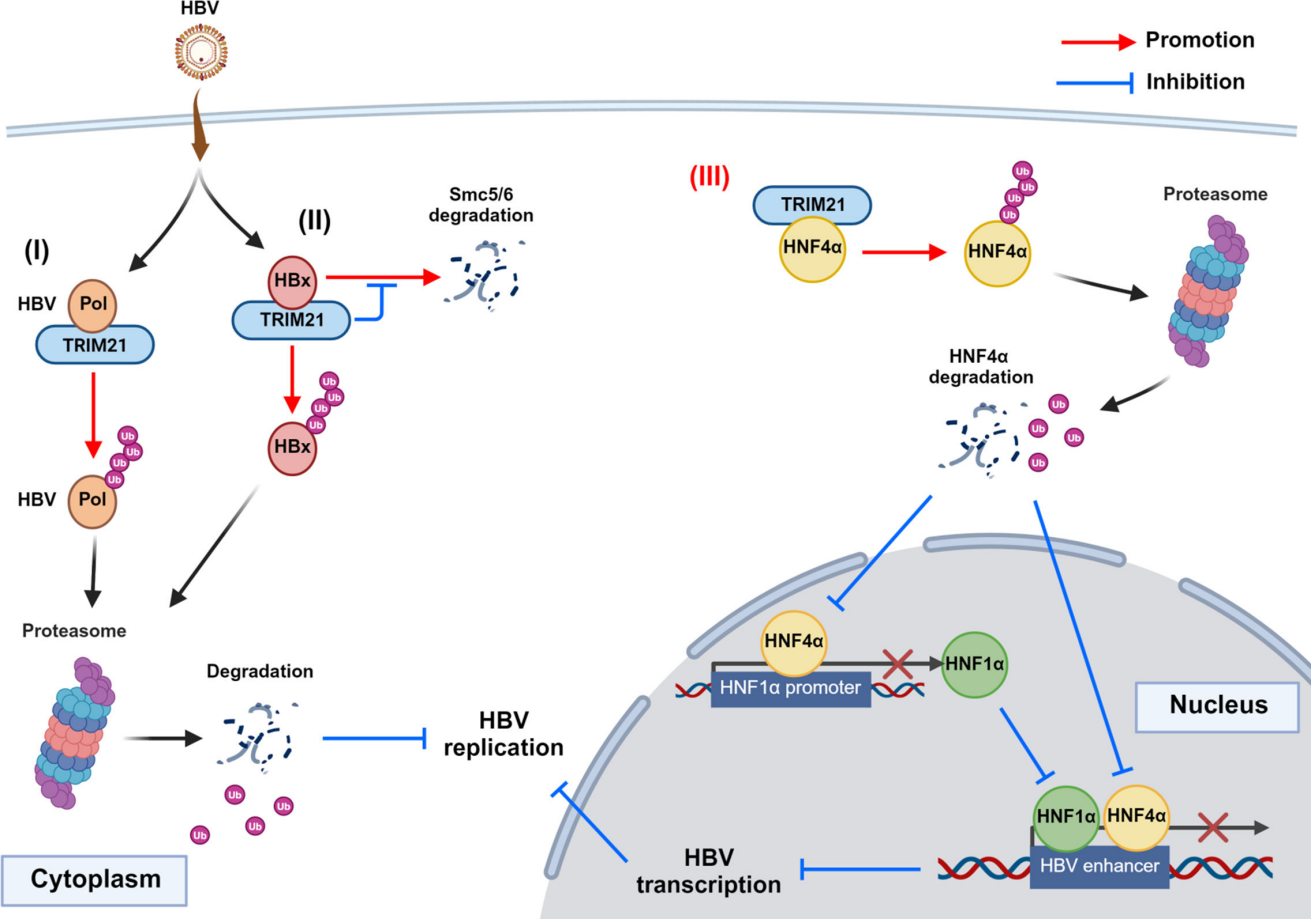
A hypothetical model of TRIM21 as an HBV host restriction factor. (**I**) The E3-ubiquitin ligase TRIM21 inhibited HBV replication through the degradation of HBV polymerase, an essential factor for the reverse transcription of HBV from pregenomic RNA (42). (II) Additionally, TRIM21 promotes the proteasomal degradation of HBx, which decreases Smc5/6 complex as a repressor for HBV transcription on cccDNA (24). (III) In this study, the novel E3-ubiquitin ligase function of TRIM21 inhibits HBV replication through the down-regulation of both HNF4α and HNF1α, key regulators of cccDNA transcription. TRIM21 binds to HNF4α, for ubiquitination leading to proteasomal degradation. The degradation of HNF4α, an activator for the promoter of HNF1α, leads to the down-regulation of HNF1α transcription. The loss of essential transcriptional factors of HBV enhancers (HNF4α and HNF1α) results in the reduction of HBV transcription and replication.

TRIM21 has been known as a critical regulator in the signaling pathway of type I IFNs during viral infection. The RING domain of TRIM21 induces the poly-ubiquitination of MAVS to positively regulate interferon regulatory factor 3 (IRF3)-mediated innate immune responses (36, 37). TRIM21 can bind to the antibody-virus complex to promote an indirect antibody-dependent intracellular neutralization (ADIN) (38, 39). In a previous study, a developed antibody targeting intracellular HBx inhibits HBV through the TRIM21-dependent ADIN pathway (23). Additionally, previous studies uncovered the role of the RING domain in ubiquitination-dependent proteasomal degradation of viral proteins. TRIM21 interacts with severe acute respiratory syndrome coronavirus 2 (SARS-CoV-2) nucleocapsid for ubiquitination to suppress the virus (40). The nucleocapsid of the porcine epidemic diarrhea virus (PEDV) can also be ubiquitylated by TRIM21, thus inhibiting PEDV proliferation (41). Especially for HBV, TRIM21 promotes HBV DNA polymerase degradation through poly-ubiquitination using the RING domain to suppress HBV replication (Fig. 9 I) (42). E3-ubiquitin ligase activity of the RING domain participates in the poly-ubiquitination of HBx, acting as a repressor of Smc5/6 complex, resulting in proteasomal degradation [Fig. 9 (II)] (24). In our findings, TRIM21 promoted the degradation of HNF4α, which is a master activator of HBV enhancers, through poly-ubiquitination with the RING domain. Degradation of HNF4α, as a transactivator of the HNF1α promoter, led to the down-regulation of HNF1α protein expression. Resultantly, the decrease in the two activators of HBV enhancers led to a reduction in HBV transcription, thereby inhibiting HBV replication [Fig. 9 (III)].

TRIM21 can inhibit HBV replication by restoring Smc5/6 complex, the HBV transcription suppressor, through HBx degradation (24). In the current study, the reduction of HBx protein by overexpression of TRIM21 was confirmed in PHHs as well (Fig. 8H). To rule out the effect of HBx, we used the HBx-defective HBV plasmids. Interestingly, overexpression of TRIM21 reduced HBV replication in the absence of HBx (Fig. 1D through K). In addition, TRIM21 reduced the activity of HBV enhancers under HBx-deficient conditions (Fig. 3). These results imply that TRIM21 inhibits HBV transcription by regulating the HNF levels.

In our previous study, we uncovered that the expression of cleaved cellular FLICE inhibitory protein induced by TNF-α suppresses HBV by inhibiting the transcription of HNF1α, HNF4α, and HNF3β through regulation of the MAPK signaling pathway (8). Also, cytokine-inducible intracellular interleukin-32γ down-regulates the transcription of HNF1α and HNF4α through control of the MAPK signaling pathway to inhibit HBV (28). We tested whether HNF4α and HNF1α expression decreasing is due to any control of the MAPK signaling pathway by TRIM21. There was no significant activation or change in factors in the MAPK signaling (Fig. 5B). Additionally, HNF4α mRNA level was not affected by TRIM21. These data support that TRIM21 can reduce the expression of HNF4α through proteasome-dependent degradation. HNF4α is known as the main activator of HNF1α expression through binding to the HNF1α promoter (43). The decrease in HNF1α expression (Fig. 5B) is indirectly inhibited by TRIM21, which is inferred to be an inhibition of transcription (Fig. 5C) due to a decrease in HNF4α expression.

The luciferase reporter assay showed that using EnhI-ΔHNF4α, the TRIM21-related attenuation in the activity of enhancer I was alleviated (Fig. 5E through G). These result supports the hypothesis that TRIM21 decreases HNF4α, thereby regulating HBV enhancer activity and reducing HBV transcription.

Various proteins involved in the TRIM family primarily interact with other proteins through the PRY-SPRY domain, which is located at the C-terminus (10, 44). For example, TRIM21 binds to the DEAD domain of DDX41 with its PRY-SPRY domain, leading to the ubiquitin-associated proteasomal degradation of DDX41 (45). TRIM21 also binds to antibody-coated pathogens within the cell via PRY-SPRY-Fc interactions (46). In this study, TRIM21 interacted with HNF4α through the PRY-SPRY domain (Fig. 6C), and in addition, HNF4α was poly-ubiquitinated via the RING domain (Fig. 6B), ultimately, leading to its degradation by proteasome (Fig. 6D and E).

Although non-stimulated endogenous TRIM21 level was barely detectable in HepG2 and Huh7 cells, it was present at basal levels, albeit not high, in PHHs (Fig. 1). This suggests that TRIM21 may act as an innate antiviral protein to combat viral infection. Interestingly, when TRIM21 mutants were overexpressed, HBV replication tended to increase significantly compared to when endogenous wild-type TRIM21 alone was present (Fig. 4C through G). This phenomenon is thought to be a negative dominant effect. These results suggest that even low expression of endogenous TRIM21 may have an effect on anti-HBV. Even though it is not known whether TRIM21 levels are directly decreased by chronic HBV, a previous study revealed that TRIM21 is down-regulated in HCC, colitis-associated cancer, and breast cancer (47–49). This reduction of endogenous TRIM21 is expected to accelerate HBV replication and synergistically leads to a poor prognosis.

In conclusion, in this study, we demonstrated a novel mechanism of IFN-γ-induced anti-HBV and showed the involvement of TRIM21 and HNF in this pathway. E3-ubiquitin ligase protein TRIM21 inhibits the expression of HNFs through promoting poly-ubiquitination of HNF4α both *in vitro* and *in vivo*. Reduction of HNFs leads to a decrease in HBV transcriptional activity, which in turn reduces HBV replication. These findings suggest a novel antiviral effect of TRIM21 in IFN-γ-induced anti-HBV and may contribute to new directions in HBV therapy.

## Data Availability

The data that support the findings of this study are available from the corresponding author upon reasonable request.
